# International Normalized Ratio (INR) instability in post-valve replacement readmissions in Sri Lanka

**DOI:** 10.1186/1749-8090-10-S1-A284

**Published:** 2015-12-16

**Authors:** Muditha N Lansakara, Mohamed N Jazeel, Imbulpitiya GP Duminda, K Gnanakanthan, Samath D Dharmaratne

**Affiliations:** 1 Department of Cardiothoracic Surgery, Teaching Hospital, Kandy, Sri Lanka; 2 Department of Community Medicine, Faculty of Medicine, University of Peradeniya, Sri Lanka and Institute for Health Metrics and Evaluation, Department of Global Health, School of Public Health, University of Washington, Seattle, WA, USA

## Background/Introduction

Anticoagulation plays an essential part of the follow up of all patients with prosthetic heart valves, who require lifelong anticoagulation therapy with monitoring of INR to avoid mechanical valve thrombosis and thromboembolism. Studies on INR monitoring are scarce in Sri Lanka.

## Aims/Objectives

To identify demography and clinical presentation of patients admitting to Cardiothoracic Unit with INR instability.

## Method

All re-admissions over a period of one and half years from October 2012 following a valve replacement with INR instability were included. An interviewer administered structured questionnaire was used for data collection. The data was analyzed using SPSS statistical software. Ethical clearance was obtained from the Ethical Review Committee of the THK.

## Results

There were 43 readmissions during the study period with INR instability of which 74.4% did not have complications (bleeding or valve dysfunction), on admission. The mean age of the sample was 46.3 years (SD = 13.4 years) and 53.5% were females. The mean weight of them was 55.3 kg (SD = 11.8kg) and the mean height 160.1 cm (SD = 15.8 cm). Of the 25.6% (N = 11) who was admitted with complications majority (N = 7,63.6) had bleeding manifestations. No one was on any other interacting drug with Warfarin at the time of data collection. Nearly half (48.8%) had Mitral (MVR), 40% Aortic (AVR) and the rest Double (DVR) valve replacements. Age (p = 0.40), height (p = 0.35) and weight (p = 0.20) were not statistically significantly different between the type of valve replacement.

## Discussion/Conclusion

The majority did not have complications and the type of the valve replaced was not statistically significantly associated with INR instability. Studying the reasons for INR instability could be important for the future management of prosthetic valve replacement patients.

